# Essential Control of the Function of the Striatopallidal Neuron by Pre-coupled Complexes of Adenosine A_2A_-Dopamine D_2_ Receptor Heterotetramers and Adenylyl Cyclase

**DOI:** 10.3389/fphar.2018.00243

**Published:** 2018-04-09

**Authors:** Sergi Ferré, Jordi Bonaventura, Wendy Zhu, Candice Hatcher-Solis, Jaume Taura, César Quiroz, Ning-Sheng Cai, Estefanía Moreno, Verónica Casadó-Anguera, Alexxai V. Kravitz, Kimberly R. Thompson, Dardo G. Tomasi, Gemma Navarro, Arnau Cordomí, Leonardo Pardo, Carme Lluís, Carmen W. Dessauer, Nora D. Volkow, Vicent Casadó, Francisco Ciruela, Diomedes E. Logothetis, Daniel Zwilling

**Affiliations:** ^1^Integrative Neurobiology Section, National Institute on Drug Abuse, Intramural Research Program, National Institutes of Health, Baltimore, MD, United States; ^2^Circuit Therapeutics, Inc., Menlo Park, CA, United States; ^3^Unitat de Farmacologia, Departament de Patologia i Terapèutica Experimental, Facultat de Medicina i Ciències de la Salut, IDIBELL, Universitat de Barcelona, Barcelona, Spain; ^4^Institut de Neurociències, Universitat de Barcelona, Barcelona, Spain; ^5^Center for Biomedical Research in Neurodegenerative Diseases Network, Department of Biochemistry and Molecular Biomedicine, Faculty of Biology, Institute of Biomedicine of the University of Barcelona, University of Barcelona, Barcelona, Spain; ^6^Eating and Addiction Section, Diabetes, Endocrinology and Obesity Branch, National Institute of Diabetes and Digestive and Kidney Diseases, Intramural Research Program, National Institutes of Health, Bethesda, MD, United States; ^7^Laboratory of Neuroimaging, National Institute on Alcohol Abuse and Alcoholism, Intramural Research Program, National Institutes of Health, Rockville, MD, United States; ^8^Department of Biochemistry and Physiology, Faculty of Pharmacy, University of Barcelona, Barcelona, Spain; ^9^Laboratory of Computational Medicine, School of Medicine, Autonomous University of Barcelona, Bellaterra, Spain; ^10^Department of Integrative Biology and Pharmacology, McGovern Medical School, University of Texas Health Science Center at Houston, Houston, TX, United States; ^11^Department of Pharmaceutical Sciences, Bouvé College of Health Sciences, Northeastern University, Boston, MA, United States

**Keywords:** striatopallidal neuron, adenosine A_2A_ receptor, dopamine D_2_ receptor, GPCR heteromers, adenylyl cyclase, caffeine, akinesia, apathy

## Abstract

The central adenosine system and adenosine receptors play a fundamental role in the modulation of dopaminergic neurotransmission. This is mostly achieved by the strategic co-localization of different adenosine and dopamine receptor subtypes in the two populations of striatal efferent neurons, striatonigral and striatopallidal, that give rise to the direct and indirect striatal efferent pathways, respectively. With optogenetic techniques it has been possible to dissect a differential role of the direct and indirect pathways in mediating “Go” responses upon exposure to reward-related stimuli and “NoGo” responses upon exposure to non-rewarded or aversive-related stimuli, respectively, which depends on their different connecting output structures and their differential expression of dopamine and adenosine receptor subtypes. The striatopallidal neuron selectively expresses dopamine D_2_ receptors (D2R) and adenosine A_2A_ receptors (A2AR), and numerous experiments using multiple genetic and pharmacological *in vitro, in situ* and *in vivo* approaches, demonstrate they can form A2AR-D2R heteromers. It was initially assumed that different pharmacological interactions between dopamine and adenosine receptor ligands indicated the existence of different subpopulations of A2AR and D2R in the striatopallidal neuron. However, as elaborated in the present essay, most evidence now indicates that all interactions can be explained with a predominant population of striatal A2AR-D2R heteromers forming complexes with adenylyl cyclase subtype 5 (AC5). The A2AR-D2R heteromer has a tetrameric structure, with two homodimers, which allows not only multiple allosteric interactions between different orthosteric ligands, agonists, and antagonists, but also the canonical Gs-Gi antagonistic interaction at the level of AC5. We present a model of the function of the A2AR-D2R heterotetramer-AC5 complex, which acts as an integrative device of adenosine and dopamine signals that determine the excitability and gene expression of the striatopallidal neurons. The model can explain most behavioral effects of A2AR and D2R ligands, including the psychostimulant effects of caffeine. The model is also discussed in the context of different functional striatal compartments, mainly the dorsal and the ventral striatum. The current accumulated knowledge of the biochemical properties of the A2AR-D2R heterotetramer-AC5 complex offers new therapeutic possibilities for Parkinson’s disease, schizophrenia, SUD and other neuropsychiatric disorders with dysfunction of dorsal or ventral striatopallidal neurons.

## Introduction

One of the most noticeable functions of adenosine in the brain is the ability to impose a brake on the central dopaminergic system. This inhibitory role of adenosine is largely mediated by the activation of one subtype of adenosine receptor, the A_2A_ receptor (A2AR), particularly expressed by one type of neuron localized in the striatum, the striatopallidal neuron. The striatum is the brain area with the highest innervation of dopamine and the highest expression of dopamine receptors in the brain (Gerfen, 2004), and the striatopallidal neuron expresses the highest density of A2AR and dopamine receptors of the D_2_ subtype (D2R) than any other neuron in the central nervous system (Gerfen, 2004; Schiffmann et al., 2007). It is now well accepted that adenosine controls the function of the striatopallidal neuron through intermolecular interactions between A2AR and D2R, with the formation of receptor heteromers.

Since its initial discovery, now more than 25 years ago (Ferré et al., 1991b), A2AR-D2R interactions have become a model for the study of allosteric interactions within G protein-coupled receptor (GPCR) heteromers, with the emergence of a new concept: allosteric interactions between orthosteric ligands (reviewed in Ferré et al., 2014). But recent findings indicate that the A2AR-D2R heteromer will also become a model for receptor-receptor interactions previously thought to take place downstream, on converging signaling molecules, which were often labeled as “interactions at the second messenger level.” The antagonistic canonical interaction at the level of adenylyl cyclase (AC), between a Gs/olf-coupled receptor, such as the A2AR, and a Gi/o-coupled receptor, such as the D2R, represents a classical example. Thus, a recent study demonstrates that this canonical interaction is dependent on the oligomerization of A2AR and D2R and the AC subtype AC5 (Navarro et al., 2018). This discovery implies that the striatal A2AR-D2R heteromer could explain most pharmacological effects of A2AR and D2R ligands, with implications for various neuropsychiatric disorders.

The understanding of the role of striatal adenosine and A2AR-D2R heteromers in striatal function and dysfunction will be here revisited within the framework of, not only the new developments on A2AR-D2R heteromers, but also most recent developments on the function of different striatal compartments and striatal dopamine, particularly on the function of the striatopallidal neuron. First, we summarize the current knowledge of the role of dopamine in the different striatal compartments. Next, we analyze the role of adenosine-dopamine interactions in the modulation of the function of the striatopallidal neuron. We then propose a functional model for the A2AR-D2R heterotetramer-AC5 complex in the striatopallidal neuron, a complex formed by one A2AR homodimer coupled to a Golf protein, a D2R homodimer coupled to a Gi protein and its signaling molecule AC5. The model is then used to reevaluate the pharmacological effects of adenosine receptor ligands, including caffeine. Finally, it is proposed that A2AR-D2R heterotetramer-AC5 complexes localized in striatopallidal neurons can be used as targets for the treatment of neuropsychiatric symptoms, such as akinesia and apathy. We also present new results of the effect of the A2AR antagonist SCH 442416 in radioligand binding, locomotor activation and optogenetic experiments in mice, which reconcile previous studies with the same compound that were apparently incompatible with our hypothesis of a predominant population of striatal A2AR-D2R heteromers that modulate striatopallidal neuronal function.

## Functional Distinction of Striatal Compartments

**FIGURE 1:**
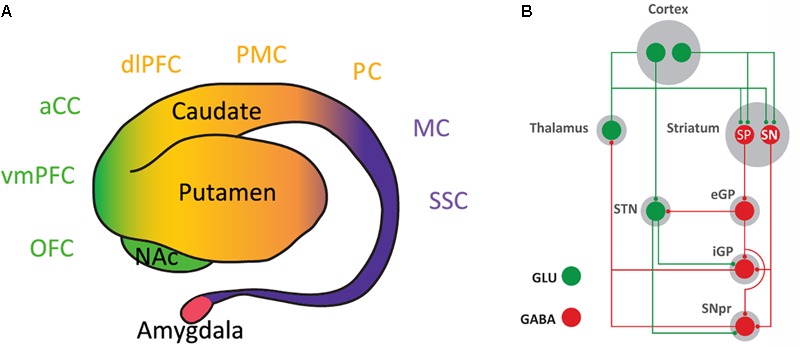
Inputs and outputs of the striatum. **(A)** Lateral view of the striatum and amygdala (of human and non-human primates). The classical morphological subdivision of striatal compartments in nucleus accumbens (NAc), caudate and putamen nuclei can be functionally classified according to the cortical inputs in: ventral striatum (in green), which receives inputs from the ventromedial prefrontal cortex (vmPFC), orbitofrontal cortex (OFC), and anterior cingulate cortex (aCC); rostral-dorsal striatum (in orange), which receives inputs from frontal and parietal association areas, such as the dorsolateral prefrontal cortex (dlPFC), premotor cortex (PMC), and parietal cortex (PC); and caudal-rostral striatum (in purple), which receives inputs from the primary motor cortex (MC) and the somatosensory cortex (SSC). **(B)** Basal ganglia circuitry. The striatonigral neuron (SN) directly connects the striatum with the output structures of the basal ganglia: the internal segment of the globus pallidus (iGP) and the substantia nigra pars reticulata (SNpr). The striatopallidal neuron (SP) connects with the output structures by relays in the external segment of the globus pallidus (eGP) and the subthalamic nucleus (STN); GLU, glutamate.

The striatum is the main input structure of the basal ganglia. Although in humans and non-human primates it has been classically subdivided into nucleus accumbens (NAc), caudate and putamen, it can be functionally subdivided in three different compartments: ventral, rostral-dorsal and caudal-dorsal striata (**Figure 1A**). The ventral striatum concept has expanded from its initial inclusion of areas innervated by the dopaminergic cells of the ventral tegmental area (VTA), mostly the NAc with its two compartments core and shell and the olfactory tubercle, to the striatal areas receiving glutamatergic inputs from the ventromedial prefrontal cortex, orbitofrontal cortex and anterior cingulate cortex (Haber and Behrens, 2014) (**Figure 1A**). In fact, the orbitofrontal cortex and the anterior cingulate cortex, respectively, receive partial and predominant dopaminergic innervation from the substantia nigra pars compacta (SNpc; Haber and Behrens, 2014). Furthermore, the ventral striatum receives afferent glutamatergic projections from the insular cortex, amygdala and hippocampus (Haber and Behrens, 2014). The rostral-dorsal striatum receives glutamatergic input from frontal and parietal association areas and the caudal-dorsal striatum from the primary motor and somatosensory cortices (**Figure 1A**). Both rostral-dorsal and caudal-dorsal striata receive their dopaminergic input from the SNpc (Haber, 2014; Haber and Behrens, 2014).

The ventral striatum forms part of decision-making brain circuits involved in reward valuation tasks, which determine and store reward values (often named as “subjective values of rewards”) and constantly choose the reward to be obtained by a process of maximizing utilities associated with different options, the highest benefit/cost ratio (Kable and Glimcher, 2009). ‘Delay discounting’ (DD), ‘effort discounting’ (ED), and ‘low-probability discounting’ (LPD) refer to the empirical finding that both humans and animals value immediate, low-effort and high probability rewards more than delayed, high-effort and low probability rewards. A large number of behavioral and clinical studies indicate that DD, ED, and LPD are independent variables possibly involving corticostriatal circuits with different ventral striatal compartments differentially connected to different prefrontal cortical areas (Prévost et al., 2010; Stopper and Floresco, 2011). A main role of the ventral striatum, classically labeled as an interface between motivation and action (Mogenson et al., 1980), can be synthesized as determining “whether to respond” while that of the dorsal striatum is “how to respond” to reward-associated stimuli (Ferré, 2017). All reward-related, dopamine-dependent functions, including the facilitation of reward-oriented behavior and learning of stimulus-reward and reward-response associations (positive reinforcement; Wise, 2004), are simultaneously processed by the rostral-dorsal and caudal-dorsal striata. In relation to positive reinforcement, the rostral-dorsal striatum is predominantly involved in an initial, more controlled (contingent on the outcome) and less permanent learning, while the caudal-dorsal striatum is involved in a slower, less controlled (non-contingent on the outcome) but long-lasting learning (Kim and Hikosaka, 2015). The same functional dichotomy, “volitional” and “automatic” learning, but with a medial-lateral distribution, can also be demonstrated in the rodent striatum (Voorn et al., 2004; Yin and Knowlton, 2006; Balleine and O’Doherty, 2010). However, it is becoming increasingly evident that dopaminergic mesencephalic cells also process aversive-related and non-rewarded stimuli and are involved in negative reinforcement. Most dopamine cells respond by decreasing their activity upon exposure to aversive stimuli and to omission of expected rewards. Dopaminergic cells, therefore, code for positive and negative reward prediction errors, increasing their firing upon presentation of reward-related stimuli or better than expected rewards or with the termination of aversive-related stimuli and decreasing their firing upon omission of reward-related stimuli or presentation of a worse than expected reward (Steinberg et al., 2013; Chang et al., 2016).

## The A2AR-D2R-Expressing Striatopallidal Neuron

In the striatum, glutamatergic and dopaminergic inputs converge in the dendritic spines of the GABAergic medium-sized spiny neurons, which constitute more than 95% of the striatal neuronal population (Gerfen, 2004). There are two types of medium-sized spiny neurons, which define the two striatal efferent pathways that connect the striatum with the output structures of the basal ganglia, substantia nigra pars reticulata (SNpr) and internal segment of the globus pallidus (**Figure 1B**). The striatonigral neuron constitutes the *direct efferent pathway* since it connects directly with the output structures (Gerfen, 2004). The striatopallidal neuron gives rise to the *indirect efferent pathway* and connects with the pallidal complex (the external segment of the globus pallidus and the ventral pallidum), which connects with the output structures directly and through a relay in the subthalamic nucleus (Gerfen, 2004) (**Figure 1B**). While there are no apparent qualitative or quantitative differences between the glutamatergic inputs impinging on the striatonigral and striatopallidal neurons, there is a clear distinction with the receptors that process the dopaminergic signals. Thus, the striatonigral neuron expresses dopamine D_1_ receptors (D1R), a prototypical Gs/olf-coupled stimulatory receptor (Golf in the striatum), while the striatopallidal neuron expresses D2R, a prototypical Gi/o-coupled inhibitory receptor (Gerfen, 2004; Bertran-Gonzalez et al., 2008). This established scheme breaks down in the most ventral striatal compartment, the shell of the NAc. The most ventral striatopallidal neurons project to the ventromedial and ventrolateral parts of the ventral pallidum, which does not connect with the output structures or relay in the subthalamic nucleus (Root et al., 2015). Instead, these regions of the ventral pallidum represent output structures of the basal ganglia themselves, since they project to the medio-dorsal thalamus, lateral hypothalamus and lateral habenula (Root et al., 2015). Furthermore, some ventral striatopallidal neurons also express D1R, with some degree of co-localization but still largely segregated from D2R (Bertran-Gonzalez et al., 2008; Frederick et al., 2015).

A mechanism by which dopamine is directly involved with positive and negative reinforcement is emerging from the study of the functional role of the direct and indirect striatal efferent pathways. Using recently developed optogenetic techniques, it has been possible to dissect a differential role of the direct and indirect pathways in mediating “Go” responses upon exposure to reward-related stimuli and “NoGo” responses upon exposure to non-rewarded or aversive-related stimuli, respectively, which depends on their different connecting output structures and their differential expression of dopamine receptor subtypes (Hikida et al., 2010, 2013; Kravitz et al., 2010, 2012; Freeze et al., 2013; Danjo et al., 2014). The differential connectivity entails that activation of striatonigral and striatopallidal neurons lead to qualitatively different behavioral responses, with the most obvious being the respective facilitation and inhibition of psychomotor activity. At this point, following Wise and Bozarth (1987), we should make the distinction between “psychomotor” and simply “motor” responses. Psychomotor responses, which include forward locomotion or withdraw, have a characteristic dependence on external stimuli; increases or decreases of dopamine enhance or diminish the responsiveness to those stimuli, respectively. The differential affinities of D1R and D2R for endogenous dopamine and their respective predominant expression in striatonigral and striatopallidal neurons provide a fine-tuning device by which bursts and pauses of dopamine neurons can differentially influence their activity (Roitman et al., 2008; Bromberg-Martin et al., 2010; Macpherson et al., 2014). Dopamine has significantly higher affinity for D2R than for D1R. Therefore, D2R are more activated than D1R by basal dopamine levels and are more sensitive to the effects of dopamine pauses, while D1R are more sensitive to dopamine bursts, to conditions of larger dopamine release. Bursts of dopamine neurons result in large dopamine release, which predominantly increases the activation of stimulatory D1R and causes the direct pathway to promote high-value reward-associated movements, whereas the lower basal dopamine levels predominantly activate D2R, which are inhibitory and remove activation of the indirect pathway, thus suppressing low-value reward-associated or high-value punishment-associated movements (Frank, 2005; Hikosaka, 2007; Dreyer et al., 2010; Hikida et al., 2010, 2013; Kravitz et al., 2012; Danjo et al., 2014). Nevertheless, we should not ignore the fact that D2R are not completely occupied by endogenous dopamine and that bursts of dopamine are also able to enhance D2R signaling, therefore participating to the psychomotor activation guided by the stimuli associated with the concomitant increase in dopaminergic cell firing. However, strong dopamine receptor activation basically promotes potentiation of corticostriatal synapses onto the direct pathway and learning from positive outcomes (positive reinforcement), while weak dopamine receptor activation promotes potentiation of corticostriatal synapses onto the indirect pathway and learning from negative outcomes (negative reinforcement) (Frank et al., 2004; Nakamura and Hikosaka, 2006; Shen et al., 2008; Voon et al., 2010).

**FIGURE 2:**
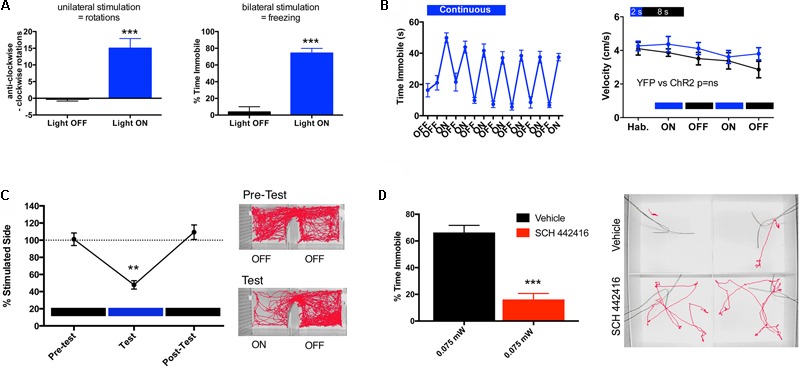
Behavioral effects of the optogenetic stimulation of the striatopallidal neuron in mice. The adeno-associated virus (AAV) containing the Cre-sensitive pAAV5-EF1a-DIO-hChR2(H134R)-YFP virus was bilaterally injected into the dorsal-medial striatum (1 μl at coordinates M-L ± 1.5, A-P + 0.8 and D-V –3.5, relative to bregma) of a BAC transgenic A2AR-Cre mouse (C57BL/6 background). Fiber-optic cannulas were implanted 0.5 μm above the injection site and the virus was allowed to incubate for 4 weeks before the start of behavioral testing. **(A)** At high intensity of stimulation, unilateral illumination results in rotational behavior and bilateral illumination results in freezing. In **(A)** left graph, mice were unilaterally illuminated (left side) with constant light at 1 mW (measured at the fiber tip) and anti-clockwise vs. clockwise (ipsilateral vs. contralateral) rotations were recorded for a duration of 5 min; results are expressed as mean ± SEM; Student’s paired *t*-test showed significant differences in the number of rotations in the illuminated condition (light ON) compared to control (light OFF) (^∗∗∗^*p* < 0.001, *n* = 4). In **(A)** right graph, mice were illuminated bilaterally with constant light at equal intensity (1 mW per side) and time spent immobile over a period of 1 min was determined; results are expressed as mean ± SEM; Student’s paired *t*-test showed significant differences between the illuminated condition (light ON) compared to control (light OFF) (^∗∗∗^*p* < 0.001, *n* = 11). **(B)** Comparison of different light illumination paradigms; bilateral circuit activation produced different effects on motor behavior depending on the pattern of light stimulation; continuous light for 1 min induced freezing and motor impairment. In **(B)** left graph, mice were bilaterally stimulated alternating 1-min light ON/OFF blocks, while freezing (expressed as time immobile) was scored through several cycles over 15 min (results are expressed as mean ± SEM, *n* = 11). In **(B)** right graph, mice were pulse stimulated using a 2-s ON/8-s OFF illumination paradigm and did not exhibit ambulatory impairment; velocity of ambulation was monitored over 3-min bins; blue and black plots represent ambulation (in mean ± SEM) of mice injected with the virus expressing ChR-YFP (*n* = 20) or YFP (control, *n* = 10), respectively; two-way ANOVA, did not show significant differences between both groups (*p* > 0.05). **(C)** Aversive behavior driven by striatopallidal neuron activation using stimulation parameters that did not produce motor impairment; in a real-time place-preference assay pulsed light illumination (2-s ON/8 s-OFF) was triggered automatically upon entry into the predesignated ‘stimulation side’ of the chamber; the amount of time spent in the light-paired chamber was quantified over 20-min blocks recorded before, during and immediately after light stimulation. In **(C)** left graph, mice demonstrated a significant light-mediated aversion, a reduction in the percentage of time (in mean ± SEM) spent in the stimulated side; analyzed statistically by one-way repeated measures ANOVA and Tukey *post hoc* test (^∗∗^*p* < 0.01; *n* = 8). In **(C)** right panel, example tracks show robust aversion to the left stimulation chamber during the test session. **(D)** A2AR antagonist reduces freezing phenotype. In **(D)** left graph, a 5-min pre-test injection of the A2AR antagonist SCH 442416 (3 mg/kg, i.p.) significantly reduced the percent time freezing (in mean ± SEM) at light power levels of 0.075 mW; Student’s paired *t*-test showed significant difference between the groups treated with and without SCH 442416 (^∗∗∗^*p* < 0.001; *n* = 11). In **(D)** right panel, example tracks showing SCH-mediated counteraction of optogenetically induced freezing. All behaviors were performed in custom-built arenas and activity monitored and automatically scored using a Noldus Ethovision video tracking system.

Another important phenotypical difference between the striatopallidal and striatonigral neurons is their differential expression of adenosine receptor subtypes. The striatopallidal neurons selectively express A2AR, in fact, the highest density in the brain (Schiffmann et al., 2007). On the other hand, A2AR are absent from the striatonigral neurons, which express adenosine A_1_ receptors (A1R) (Ferré et al., 1997). A2AR can then be used as a marker of the striatopallidal neuron. For instance, to identify the function of the striatopallidal neuron, studies using Bacterial Artificial Chromosome (BAC) transgenic mice have targeted the regulatory elements of either the D2R or the A2AR (Durieux et al., 2009; Valjent et al., 2009; Freeze et al., 2013). We used BAC transgenic mouse lines that express Cre recombinase under the control of regulatory elements of the D2R (D2R-Cre mice) or the A2AR (A2AR-Cre mice), allowing the selective expression of channelrhodopsin 2 (ChR2) by striatopallidal neurons (Kravitz et al., 2010; Freeze et al., 2013; Zwilling et al., 2014). This was achieved by bilateral injection of an adeno-associated virus (AAV) containing a Cre-sensitive vector with a double-floxed inverted open reading frame encoding a fusion of ChR2 and enhanced yellow fluorescence protein into the dorsomedial striatum. Then, fiber-optic cannulas implanted immediately above the injection site allowed the local delivery of light with the concomitant selective optogenetic activation of a large fraction of dorsal striatopallidal neurons. Unilateral optogenetic stimulation led to significant ipsilateral rotational behavior, while bilateral optogenetic stimulation led to a significant decrease in locomotor activity (Kravitz et al., 2010; Freeze et al., 2013; Zwilling et al., 2014) (**Figure 2A**). These results were opposite to those obtained by the selective ablation of a large proportion of dorsal and ventral striatopallidal neurons in BAC transgenic A2AR-Cre mice by Cre-mediated expression of a diphtheria toxin receptor and diphtheria toxin injection, which led to hyperlocomotion (Durieux et al., 2009).

Altogether, these optogenetic and genetic targeting experiments agree with the increase and decrease of “NoGo” responses upon the respective activation or inactivation of striatopallidal neurons. Hypo- or hyperlocomotion represents an outcome of the respective sustained activation or deactivation of a large number of striatopallidal neurons, which more discretely should represent the respective facilitation and inhibition of withdrawal behavior from low-value reward-associated or high-value punishment-associated movements. We addressed more directly this assumption in optogenetic experiments with A2AR-Cre mice, by selectively inducing the expression of ChR2 in dorsal striatopallidal neurons and by using more discrete parameters of stimulation (Zwilling et al., 2014). **Figure 2B** shows the comparison of two different parameters of bilateral optogenetic stimulation in the dorso-medial striatum on locomotor activity. Continuous light for 1 min induced freezing and therefore an impairment of motor activity that would interfere with the analysis of behavior in a real-time place-preference study. On the other hand, mice that were pulse-stimulated using a 2-s ON/8-s OFF illumination paradigm did not demonstrate ambulatory impairment (**Figure 2B**). When this pulse-stimulation was triggered when the mouse entered one of the chambers of a place-preference box, the animal showed a very significant aversion-like behavior to that side (**Figure 2C**) (Zwilling et al., 2014). These results also complement those obtained by Hikida et al. (2010, 2013) in experiments with selective bilateral inactivation of the dorsal or ventral striatopallidal neuron by means of doxycycline-dependent, pathway-specific expression of tetanus toxin (driven by the promoter of the gene coding the neuropeptide enkephalin, selectively expressed by striatopallidal neurons). A counteraction of the expression in addition to the acquisition of aversion-like behavior was also demonstrated by using an asymmetric design, with targeted unilateral inactivation of the ventral striatopallidal neurons with tetanus toxin and the contralateral infusion of a D2R agonist (but not a D2R antagonist or D1R agonists or antagonists) or an A2AR antagonist (Hikida et al., 2013). Similarly, we could demonstrate that the systemic administration of the A2AR antagonist SCH 442416 (3 mg/kg i.p.) significantly decreases the locomotor depression induced by low-intensity optogenetic stimulation of the dorsal-medial striatopallidal neurons (**Figure 2D**). These results would imply a significant role of an endogenous adenosinergic tone in the facilitation of the striatopallidal neuronal function mediated by A2AR. In fact, numerous neurochemical studies imply that A2AR signaling is especially involved in driving the activation of the striatopallidal neuron upon D2R disinhibition (see below), therefore in driving the suppression of the behavior associated with non-rewarded and punishment-associated stimuli.

## The A2AR-D2R Receptor Heterotetramer-AC5 Complex

There is a large amount of experimental evidence indicating the existence of a predominant striatal population of A2AR and D2R that control striatopallidal neuronal function (Ferré et al., 1993, 2016; Azdad et al., 2009; Bonaventura et al., 2015). Recent studies suggested that A2AR-D2R heteromers assemble into a heterotetrameric structure, with A2AR and D2R homodimers coupled to their respective cognate Gs (more precisely the Golf isoform) and Gi proteins (Bonaventura et al., 2015). The heterotetrameric structure would provide the frame for multiple adenosine-dopamine interactions and for interactions between exogenous A2AR and D2R ligands (Navarro et al., 2014; Ferré et al., 2016). One of the most prominent interactions is the allosteric negative effect of A2AR ligands on the affinity and efficacy of D2R ligands (*allosteric interaction*) (Ferré et al., 1991b; Azdad et al., 2009; Bonaventura et al., 2015), which has been demonstrated to depend on A2AR-D2R heteromerization by the use of synthetic peptides that selectively interfere with the heteromeric interface, both in mammalian transfected cells and in striatal tissue (Azdad et al., 2009; Bonaventura et al., 2015).

**FIGURE 3:**
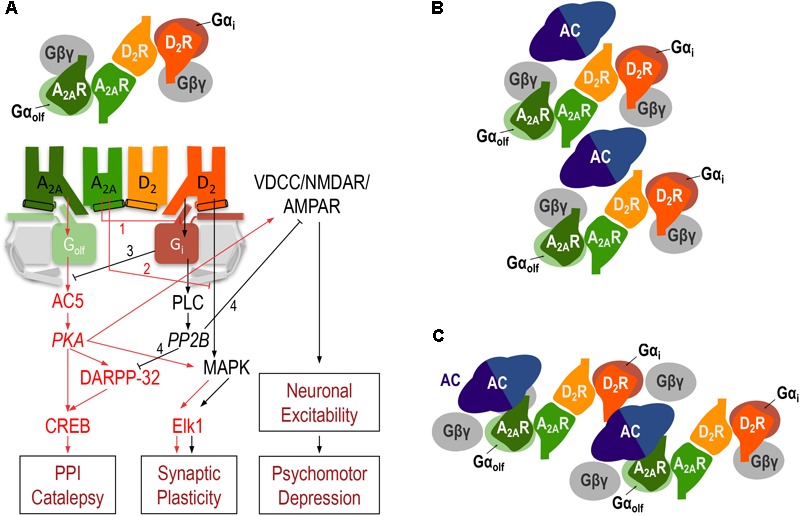
The A2AR-D2R heterotetramer. **(A)** Model representing the striatal A2AR-D2R heteromer-dependent mechanisms that modulate different biochemical and behavioral outputs. The heterotetrameric structure of the A2AR-D2R heteromer allows multiple simultaneous and reciprocal interactions between adenosine and dopamine and exogenous A2AR and D2R ligands. Mainly, the ability of adenosine or exogenous A2AR ligands to decrease G protein-dependent (1) or G protein-independent (2) signaling by dopamine or exogenous D2R ligands (allosteric interactions) and a reciprocal antagonistic interaction, the ability of D2R agonists to inhibit the A2AR agonist-mediated activation of AC5, by means of the antagonistic Gs-Gi canonical interaction at the AC5 level (3). When uninterrupted by the canonical interaction, A2AR signals through activation of AC5 and protein kinase A (PKA) with phosphorylation of ‘dopamine- and cAMP-regulated phosphoprotein of molecular weight 32,000’ (DARPP-32), which facilitates PPI and catalepsy, and voltage-dependent Ca^2+^ channels (VDCC), NMDA and AMPA receptors, resulting in an increase in the excitability of the striatopallidal neuron. When uninterrupted by the allosteric interaction, D2R signals through PLC, which leads to activation of calcineurin (PP2B). PP2B dephosphorylates PKA substrates, DARPP-32, VDCC, NMDA and AMPA receptors, providing a downstream additional mechanism of D2R-mediated inhibition of A2AR signaling (4) and leading to a decrease in the excitability of the striatopallidal neuron, which facilitates psychomotor activation. A2AR and D2R activation can also modify gene expression through different mechanisms, including G protein-dependent and independent MAPK activation and activation of the transcription factor Elk-1 (see text). In **(B,C)**, schematic slice-representation viewed from the extracellular side of the minimal functional unit of the A2AR-D2R heterotetramer in complex with AC5 (see text), in the absence **(B)** and presence **(C)** of agonists, which induce a rearrangement of the heterotetramer-AC5 interfaces (modified from Navarro et al., 2018).

In addition to the allosteric interaction, a strong reciprocal antagonistic interaction, with the ability of D2R agonists to inhibit A2AR agonist-mediated activation of AC, was first identified in mammalian transfected cells (Kull et al., 1999; Hillion et al., 2002) and more recently characterized in striatal cells in culture (Navarro et al., 2014). This represents an antagonistic Gs-Gi *canonical interaction*, the ability of an activated Gi-coupled receptor to inhibit a Gs-coupled receptor-mediated activation of AC. The A2AR-D2R canonical interaction was first observed *in situ*, in the striatum. The evidence came initially from experiments that demonstrated that the increase in the expression of the immediate-early gene *c-fos* in the striatopallidal neurons upon treatment with D2R antagonists or acute dopamine depletion could be counteracted by blocking A2AR signaling (Boegman and Vincent, 1996; Svenningsson et al., 1999). This A2AR signaling is initiated by the second messenger cyclic-AMP (cAMP), the product of AC activation. The cascade includes protein kinase A (PKA) activation, with phosphorylation of the cAMP-response element binding protein (CREB), a mechanism which is amplified by the PKA-dependent phosphorylation of ‘dopamine- and cAMP-regulated phosphoprotein of molecular weight 32,000’ (DARPP-32) (Svenningsson et al., 1998; Kull et al., 1999) (**Figure 3A**). A2AR-mediated activation of PKA also promotes phosphorylation of voltage dependent Ca^2+^ channels (VDCC), NMDA, and AMPA receptors (Håkansson et al., 2006; Azdad et al., 2009; Higley and Sabatini, 2010), which determines their degree of activation and, therefore, the degree of excitability of the striatopallidal neurons, which determines the degree of psychomotor depression (**Figure 3A**).

Activation of the D2R, when uninterrupted by co-activation of the A2AR (allosteric interaction), can also signal through phospholipase C (PLC) by a Gßγ subunit-dependent mechanism, which induces the release of inositol (1,4,5)-triphosphate (IP3), a second messenger that causes the release of intracellular Ca^2+^. This, in turn results in the subsequent activation of the Ca^2+^/calmodulin-dependent protein phosphatase calcineurin (also called protein phosphatase 2B or PP2B) (Hernandez-Lopez et al., 2000; Azdad et al., 2009) (**Figure 3A**). Phosphorylated forms of VDCC, NMDA, and AMPA receptors and DARPP-32 are main targets of PP2B. Therefore, activation of PP2B leads to a decreased neuronal excitability and represents a downstream additional mechanism of D2R-mediated inhibition of A2AR signaling (Lindskog et al., 1999) (**Figure 3A**). In addition, A2AR and D2R activation can modify gene expression through respective G protein-dependent and independent mechanisms of MAPK activation, which plays a predominant role in the mailing of signals from the synapse to the nucleus by directly activating the constitutive transcription factor Elk-1 (Besnard et al., 2011) (**Figure 3A**). Our previous work indicates that the outcome of co-activation of striatal A2AR and D2R on MAPK activation depends on the intracellular levels of Ca^2+^, which determines the binding of two different neuronal Ca^2+^-binding proteins, NCS-1 and calneuron-1 (Navarro et al., 2014). NCS-1 and calneuron bind to the A2AR-D2R heteromer upon low and high concentrations of Ca^2+^, respectively. Binding of calneuron specifically alters the ability of A2AR ligands to allosterically modulate the GTP-independent D2R ligand-mediated MAPK activation, while binding of NCS-1 also counteracts the A2AR-mediated allosteric modulation of D2R-ligand-mediated G protein signaling (and therefore the canonical interaction). This provides a mechanism by which co-activation of A2AR and D2R in the heteromer promotes and counteracts MAPK activation upon low and high concentrations of Ca^2+^, respectively (Navarro et al., 2014).

The question is how two apparently simultaneous reciprocal interactions between A2AR- and D2R signaling (allosteric and canonical interactions) can take place in the same cell. Based on some studies obtained with the A2AR antagonist SCH 442416, we initially hypothesized the existence of two populations of A2AR in striatopallidal neurons (Orrú et al., 2011b). One population would be forming heteromers with D2R and would mediate the allosteric interaction, while another population would not be forming heteromers with D2R and would allow the antagonistic interaction at the second messenger level, cAMP (Ferré et al., 2011; Orrú et al., 2011b). However, we recently hypothesized that the putative heterotetrameric structure of the A2AR-D2R heteromer could sustain both the allosteric and the canonical interactions (Navarro et al., 2014). In addition, based on the emergent view that considers GPCR homodimers as main functional units (Ferré et al., 2014), we postulated that heteromers are constituted by different interacting homodimers (Ferré et al., 2014; Ferré, 2015). This could be of special functional importance with heteromers formed by one homodimer coupled to a Gs/olf protein and another different homodimer coupled to a Gi/o protein. Our hypothesis was that such a GPCR *heterotetramer* would be part of a pre-coupled macromolecular complex that also includes AC5, the predominant AC subtype in the striatum (Lee et al., 2002), a necessary frame for the canonical antagonistic interaction at the AC level (Ferré, 2015). In fact, in mammalian transfected cells, using synthetic peptides with amino acid sequences of all transmembrane domains (TM) of A2AR and D2R and the putative TMs of AC5, we recently provided clear evidence for the existence of functional pre-coupled complexes of A2AR and D2R homodimers, their cognate Golf and Gi proteins and AC5 (Navarro et al., 2018). We first identified a symmetrical TM 6 interface for the A2AR and D2R homodimers and a symmetrical TM 4/TM 5 A2AR-D2R heteromeric interface. Computational analysis provided one minimal solution, a linear arrangement with two internal interacting A2AR and D2R protomers and two external non-interacting A2AR and D2R protomers which interact with the α-subunits of the corresponding Golf and Gi proteins (**Figure 3**). Second, we found asymmetrical interfaces formed by TMs of the receptors and putative TMs of AC5 which rearrange upon agonist exposure. Computational analysis indicated the existence of a minimal functional complex formed by two A2AR-D2R heterotetramers and two AC5 molecules (**Figures 3B,C**). In fact, this quaternary structure suggests the possible formation of zig-zagged arranged high-order oligomeric structures, a higher-order linear arrangement of GPCR heteromers and effectors (Navarro et al., 2018). Finally, we could demonstrate that this macromolecular complex provides the sufficient but necessary condition for the canonical Gs-Gi interactions at the AC level (Navarro et al., 2018). The most demonstrative experiment was that destabilization of the quaternary structure of the A2AR-D2R heterotetramer, with interfering synthetic peptides with the amino acid sequence of the TMs involved in heteromeric interface, blocked the ability of a D2R agonist to counteract AC5 activation by an A2AR agonist in striatal neurons in culture (Navarro et al., 2018).

The A2AR-D2R heterotetramer therefore acts as an integrative molecular device, which allows reciprocal antagonistic interactions between adenosine and dopamine to facilitate a switch in the activation-inhibition of the striatopallidal neuron: A preferential A2AR vs. D2R activation leads to an increase in neuronal activity determined by the A2AR-mediated activation of the AC5/PKA pathway, which is potentiated by the allosteric counteraction of D2R signaling (“1” and “2” in **Figure 3A**); a preferential D2R vs. A2AR activation leads to a decrease in neuronal activity by activation of the PLC/PP2B pathway and switching off the A2A-mediated activation of AC5 through the canonical interaction (“3” in **Figure 3A**), which we have shown depends on the integrity of the A2AR-D2R heterotetramer-AC5 complex (Azdad et al., 2009; Higley and Sabatini, 2010; Navarro et al., 2014, 2018; Bonaventura et al., 2015; Ferré, 2016; Ferré et al., 2016).

The heterotetrameric structure of the A2AR-D2R heteromer provides the framework for allosteric mechanisms of A2AR ligands that could explain recent experimental findings apparently incompatible with classical pharmacology, such as the agonist-like behavior of A2AR antagonists, which includes caffeine, a non-selective adenosine receptor antagonist. The initial unexpected finding came from a human PET study. In this study, the acute administration of caffeine produced an increase in the binding of [^11^C]raclopride, a D2R antagonist, in the putamen and ventral striatum (Volkow et al., 2015). As a significant additional finding, the caffeine-dependent increase in D2R antagonist binding in the ventral striatum correlated with an increase in alertness (Volkow et al., 2015). Considering that previous studies demonstrated antagonistic allosteric interactions between A2AR and D2R agonists, caffeine should have induced the opposite effect, a decrease in [^11^C]raclopride binding, due to an increase in the affinity of endogenous dopamine. We therefore studied the possibility of a direct allosteric modulation of caffeine on D2R agonist binding. Both the A2AR agonist CGS 21680 and caffeine significantly decreased the binding of the D2R agonist [^3^H]quinpirole in membrane preparations from sheep striatum and mammalian cells transfected with A2AR and D2R. We could also demonstrate that both agonist-agonist and antagonist-agonist allosteric modulations were dependent on heteromerization, since they were not observed when transfecting a mutated A2AR with impaired ability to heteromerize with D2R (Bonaventura et al., 2015). Therefore, we initially assumed that the caffeine-induced increase in [^11^C]raclopride binding demonstrated in PET experiments could be explained by a caffeine-induced decrease in the affinity of endogenous dopamine. However, the observation that both A2AR agonists and A2AR antagonists can produce the same allosteric interaction in the A2AR-D2R heteromer, a reduction in the affinity of agonists for the D2R, contradicts the hypothesis of a key role of allosteric interactions within the A2AR-D2R heteromer as a main mechanism involved in the opposite behavioral effects of A2AR agonists and antagonists (Ferré, 2008, 2016). Nevertheless, a biphasic effect was observed when analyzing the effect of increasing concentrations of caffeine or the selective A2AR antagonists SCH 58261 and KW 6002 on the ability of a single concentration of CGS 21680 to decrease [^3^H]quinpirole binding. Low concentrations of caffeine and the A2AR antagonists significantly counteracted the effect of CGS 21680, while high concentrations further decreased [^3^H]quinpirole binding (Bonaventura et al., 2015). Therefore, the results implied that orthosteric A2AR agonists and antagonists produce the same allosteric modulation of D2R agonist binding within the A2AR-D2R heteromer when applied individually, but, when co-applied, they cancel out each other’s effect. This could be explained by the existence of two A2AR protomers in the A2AR-D2R heteromer and allosteric interactions between orthosteric agonists and antagonists, by which simultaneous occupation of the A2AR homodimer by an agonist and an antagonist would “freeze” the ability of either ligand to allosterically modulate D2R agonist binding. The existence of allosteric interactions between orthosteric A2AR agonists and antagonists could be confirmed through binding kinetics experiments with the A2AR antagonist [^3^H]ZM 241385. Thus, when analyzing the effect of CGS 21680, caffeine and SCH 58261, only CGS 21680 modified the dissociation rate of [^3^H]ZM 241385 (Bonaventura et al., 2015). Considering that CGS 21680 and [^3^H]ZM 241385 bind to the same orthosteric site (Lebon et al., 2011), the effect of CGS 21680 could be explained by co-occupation of both ligands of the two orthosteric sites in an A2AR homodimer.

The same allosteric effects on D2R agonist binding demonstrated by A2AR agonists and antagonists were also shown in functional experiments. By measuring ERK1/2 phosphorylation in transfected cells, we could demonstrate that CGS 21680 counteracts quinpirole-induced MAPK activation, that this effect of CGS 21680 can be counteracted by low concentrations of caffeine or the A2AR antagonist SCH 58261 and that high concentration of the antagonists induce the opposite effect (Bonaventura et al., 2015). We should therefore expect that in the experimental animal A2AR antagonists behave as A2AR agonists under specific conditions. In fact, in patch-clamp experiments, we could demonstrate that the A2AR antagonist SCH 58261 counteracts the D2R antagonist-like properties of CGS 21680, but it reproduces the effect of the A2AR agonist when administered alone (Bonaventura et al., 2015). These results challenge the traditional view of competitive antagonism as the mechanism of the psychostimulant effects of caffeine (and selective A2AR antagonists). According to our model, the psychostimulant effect of caffeine can be explained by the counteraction of the allosteric interaction by co-occupation of the A2AR homodimer with caffeine and endogenous adenosine in the A2AR-D2R heterotetramer.

However, these allosteric interactions between A2AR agonists and antagonists and D2R agonists do not yet explain the increase in striatal [^11^C]raclopride binding in human PET experiments induced by caffeine. Again, counteraction by caffeine of the inhibitory effect of endogenous adenosine on the binding of endogenous dopamine should lead to a decrease of [^11^C]raclopride binding. It was then demonstrated that CGS 21680 and caffeine also inhibit the binding of [^3^H]raclopride binding in membrane preparations from striatum or transfected cells (Bonaventura et al., 2015). That these results imply allosteric interactions within the A2AR-D2R heteromer was demonstrated by their disappearance upon transfection of a mutated A2AR with impaired ability to heteromerize with D2R and by using a synthetic peptide that disrupts A2AR-D2R heteromerization (Bonaventura et al., 2015). Therefore, within the A2AR-D2R heteromer, any orthosteric A2AR ligand, agonist or antagonist, exerts a negative allosteric modulation on the affinity of any orthosteric D2R ligand, agonist or antagonist. Finally, the same as with [^3^H]quinpirole binding, we could also demonstrate a biphasic effect of caffeine on CGS 21680-mediated decrease of [^3^H]raclopride binding (Bonaventura et al., 2015). These results would at last provide a plausible mechanism for the effect of caffeine on [^11^C]raclopride binding in humans, by its ability to antagonize the effect of endogenous adenosine on the binding of the exogenous D2R antagonist. An alternative explanation could still be that caffeine blocks an adenosine-mediated internalization of A2AR-D2R heteromers (Hillion et al., 2002; Huang et al., 2013), thus leading to higher D2R availability along with higher [^11^C]raclopride binding. The positive association between caffeine-induced increases in D2R availability and caffeine-induced increases in alertness (Volkow et al., 2015) would support this interpretation, since increased D2R signaling contributes to alertness (Isaac and Berridge, 2003). Irrespective of the mechanism involved, the effect of caffeine on [^11^C]raclopride binding in human PET experiments implies its dependence on the A2AR-D2R heteromer and, therefore, that a significant proportion of striatal [^11^C]raclopride binding visualized with PET labels A2AR-D2R heteromers. Furthermore, these results call for the need to control caffeine intake when evaluating the effect of D2R ligands in humans, not only when using them as probes for imaging studies, but also when using them as therapeutic agents in neuropsychiatric disorders.

**FIGURE 4:**
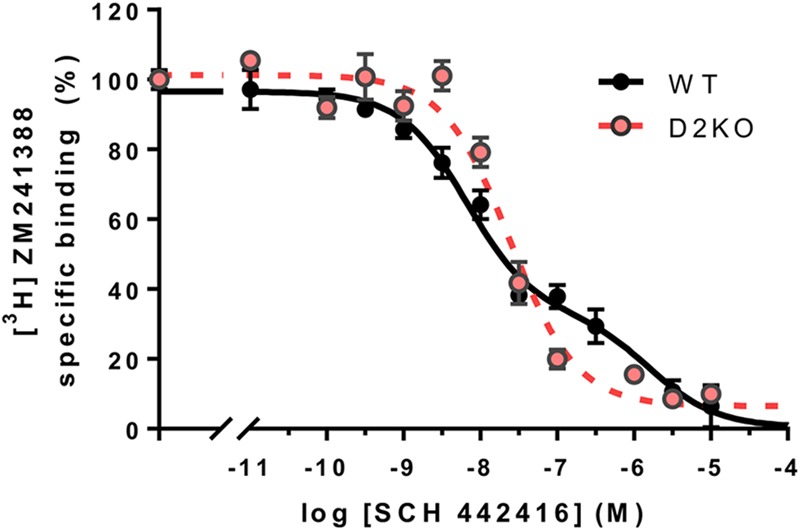
Specific A2AR-D2R heteromer-dependent properties of SCH 442416 in mouse striatum. Transgenic conditional knockout striatopallidal neuron-Drd2-KO mice were generated by crossing mice expressing Cre driven by regulatory elements of the A2AR gene (Adora2a) [B6.FVB(Cg)-Tg(Adora2a-Cre)KG139Gsat/Mmucd; GENSAT; 036158-UCD] with mice carrying conditional D2R gene (Drd2) null alleles B6.129S4(FVB)-Drd2tm1.1Mrub/J,JAX020631 (Bello et al., 2017). Membrane preparations from the striatum of striatopallidal-Drd2-KO (red) and their CRE negative littermates (WT, black) were incubated with [^3^H]ZM 241385 (2 nM) and increasing concentrations of SCH 442416 as described elsewhere (Bonaventura et al., 2015). Data points were fit to the two-state dimer receptor model (Casadó et al., 2007; Ferré et al., 2014), showing a biphasic curve due to negative cooperativity of SCH 442416 in WT mice (DCB = –1.8), but a monophasic curve in the conditional D2R null mice, thus reproducing the same behavior of SCH 442416 previously demonstrated in mammalian cells co-expressing A2AR and D2R and only A2AR, respectively (Orrú et al., 2011a).

Very different qualitative differences between several A2AR antagonists emerged when evaluating their potencies and efficacies on different *in vitro* and *in vivo* techniques. Particularly significant was the demonstration of different binding properties of the A2AR antagonist SCH 442416 depending on the presence and absence of D2R, when forming or not forming heteromers with D2R (Orrú et al., 2011a). In cells expressing A2AR and D2R, competitive-inhibition curves of the A2AR antagonist [^3^H]ZM 241388 binding vs. increasing concentrations of SCH 442416 were clearly biphasic. On the other hand, in cells expressing only A2AR, or A1R and A2AR, the curves were monophasic. When analyzing the radioligand binding experiments with the two-state dimer model (Casadó et al., 2007; Ferré et al., 2014), the data indicated a negative cooperativity of SCH 442416 binding to the A2AR (Orrú et al., 2011b; Ferré et al., 2014), an additional demonstration of A2AR homomerization. This, in fact, was the first indication that the A2AR-D2R comprises at least two A2AR protomers, in agreement with a tetrameric structure of the A2AR-D2R heteromer. We have now been able to reproduce these findings in striatal tissue, comparing the results of competitive-inhibition experiments of [^3^H]ZM 241388 binding vs. increasing concentrations of SCH 442416 in striatal membrane preparations of wild-type (WT) and striatal D2R knockout mice with a CRE-mediated deletion of D2R expression in A2AR-expressing neurons. The same as with mammalian transfected cells, the curves were biphasic or monophasic in the presence or absence of the D2R, respectively (**Figure 4**). The demonstration of the same binding properties of SCH 442416 in striatal tissue than in A2AR-D2R transfected cells implies that the A2AR-D2R heterotetramer represents the predominant population of A2AR and D2R in the striatum. Nevertheless, as mentioned below, striatal A2AR are also localized presynaptically, in glutamatergic terminals, where most probably do not form heteromers with D2R. These receptors, although playing a significant role in the modulation of striatal glutamate release, represent a very small fraction of the total of striatal A2AR, as compared to the postsynaptic A2AR.

## Revisiting the Behavioral Effects of Adenosine Receptor Ligands in the Frame of One Main Population of Striatal A2AR and D2R Forming Heteromers

**FIGURE 5:**
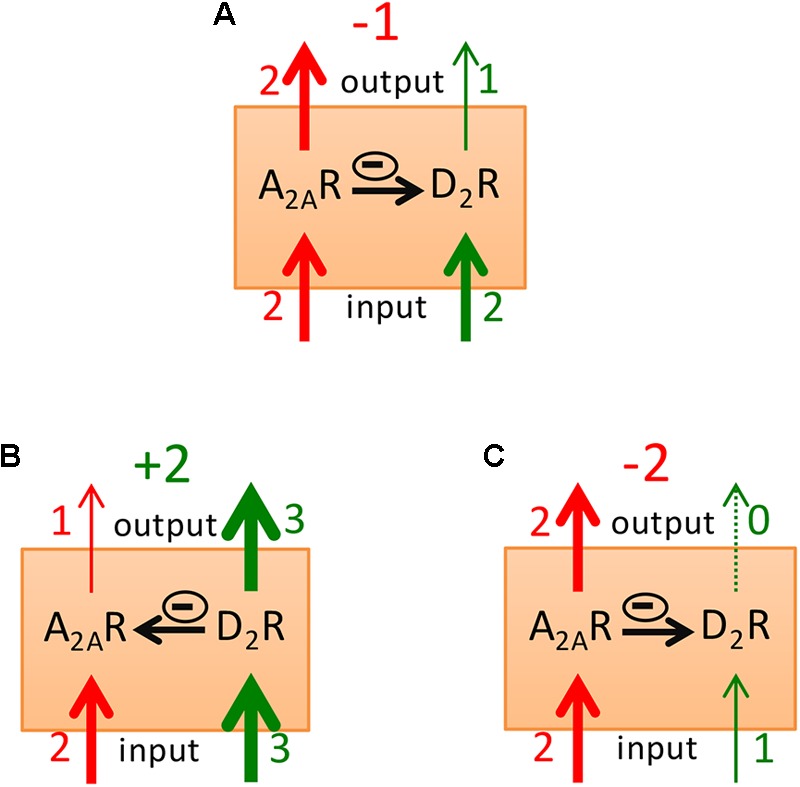
Model of the striatal A2AR-D2R receptor heteromer as a main modulator of the excitability of the striatopallidal neuron. The relative thickness (and close number) of the red and green input arrows represents the degree of activation of the A2AR and the D2R that depends on the concentration of the corresponding neurotransmitter or exogenous ligands. The thickness (and close number) of the red and green output arrows represents the intensity of A2AR and D2R signaling, respectively, which depends on the input signal for each receptor and on the predominance of either the antagonistic or the canonical interaction (represented by horizontal arrows with a minus enclosed sign). Predominant psychomotor activation or depression will result when subtraction of the A2AR signaling from the D2R receptor signaling gives a positive or negative value (also in green or red, respectively). In **(A)**, resting condition. In **(B,C)**, processing of a positive or a negative reward prediction error, respectively.

Considering that increases or decreases in the activity of the GABAergic striatopallidal neuron lead to the respective opposite effect on psychomotor activity and using a model that considers the A2AR-D2R heteromer as a key modulator of striatopallidal neuronal function, we could recently explain most psychomotor effects of caffeine (Ferré, 2016). This included the enigmatic caffeine-induced rotational behavior in rats with unilateral striatal dopamine denervation (Fuxe and Ungerstedt, 1974; Herrera-Marschitz et al., 1988; Casas et al., 1989; Garrett and Holtzman, 1995) and the ability of caffeine to significantly counteract the adipsic-aphagic syndrome in rodents with 6-hydroxy-dopamine-induced or genetic-induced dopamine deficiency (Casas et al., 2000; Kim and Palmiter, 2003, 2008). According to the model, under resting conditions there is a tonic activation of A2AR and D2R by the endogenous neurotransmitters which results in a predominant A2AR vs. D2R signaling and a predominant allosteric interaction (**Figure 5A**), which results in low psychomotor activity. Reward-related stimuli and, particularly a “better than expected” rewarding stimulus (positive reward prediction error), leads to striatal dopamine release with a predominant D2R vs. A2AR signaling, potentiated by the canonical interaction (**Figure 5B**), leading to psychomotor activation. Aversive-related stimuli or a “worse than expected” rewarding stimuli (negative reward prediction error) leads to inhibition of dopamine release, to the weakest D2R and strongest A2AR signaling, which is potentiated by the canonical interaction (**Figure 5C**), leading to psychomotor arrest.

A pathogenic hallmark of akinesia in Parkinson’s disease is a pronounced hyperactivity of the striatopallidal neuron associated with the dopamine deficiency and pronounced decrease in the tonic D2R signaling. The discoveries on A2AR localization and function in striatopallidal neurons gave the rational for the recently implemented A2AR antagonists in this disease (Müller and Ferré, 2007; Morelli et al., 2009; Armentero et al., 2011). It was initially suggested that the value of A2AR antagonists as antiparkinsonian agents would depend mostly on the allosteric interaction, on the ability of A2AR antagonists to potentiate D2R signaling by concomitant administration of L-dopa or a selective D2R agonist (Ferré et al., 1991b, 1992). This was followed by behavioral studies with genetic inactivation of A2AR and D2R, which stressed the value of the canonical interaction, which was assumed to be independent of intermolecular interactions between A2AR and D2R (Chen et al., 2001). As mentioned above, the existence of the two apparently incompatible simultaneous allosteric and canonical interactions led to the hypothesis of the existence of two populations of A2AR in the striatopallidal neuron, one population forming heteromers with D2R and sustaining the allosteric interaction and another population not forming heteromers with D2R and sustaining the canonical interaction (Ferré et al., 2011; Orrú et al., 2011b). The unique pharmacological properties of SCH 442416, with its specific reduced affinity for the A2AR-D2R heteromer, due to negative cooperativity, were then exploited to attempt a pharmacological dissection of the two populations of postsynaptic A2AR. In fact, we previously used this strategy to dissect postsynaptic from presynaptic A2AR, which forms heteromers with A1R in the striatal glutamatergic terminals, where they play an important role in the modulation of glutamate release (Ciruela et al., 2006; Quiroz et al., 2009). A correlation had been shown with the higher potency of SCH 442416 to block presynaptic A1R-A2AR heteromers vs. postsynaptic A2AR-D2R heteromers and its higher potency to inhibit corticostriatal glutamate release than to produce locomotor activation (Orrú et al., 2011a). The preferential presynaptic profile of SCH 442416 was confirmed by other studies including other research groups (Hobson et al., 2013; O’Neill et al., 2014) and was suggested to provide a therapeutic approach for conditions with increased corticostriatal transmission, such as cannabinoid use disorder (Justinová et al., 2014). An apparently stronger potency of SCH 442416 to counteract locomotor depression induced by the D2R antagonist raclopride, as compared to that produced by the A2AR agonist CGS 2160, was then interpreted as the ability of SCH 442416 to also dissect the two putative postsynaptic populations of A2AR. The more sensitive population to SCH 442416 would be A2AR that do not form heteromers with D2R and that would sustain the canonical interaction (Orrú et al., 2011b). However, as mentioned before, we now know that the canonical interaction requires receptor heteromerization (Navarro et al., 2018). Therefore, we recently performed new studies on the behavioral effects of SCH 442416 upon genetic blockade of A2AR or D2R and upon administration of the A2AR agonist CGS 21680 and the D2R antagonist haloperidol, to reevaluate if they could all be explained within the framework of a predominant population of striatal postsynaptic A2AR-D2R heteromers (Taura et al., 2017).

To control strain-dependent behavioral differences and differences in drug responses, using CRISPR-Cas9 technology, we generated a D2R deficient mouse with the same genetic background as the CD-1 A2AR^-/-^ mouse (Ledent et al., 1997). CD-1 D2R^-/-^ mice showed a significant but relatively small reduction in spontaneous locomotor activity (Taura et al., 2017), as previously reported in D2R^-/-^ C57BL/6 mice (Baik et al., 1995). This is in contrast with the pronounced akinesia and catalepsy that characterize pharmacological D2R blockade (Ferré et al., 1990; Kanda et al., 1994; Shiozaki et al., 1999). Therefore, genetic D2R blockade is associated with neuroadaptations that counteract the loss of a D2R-mediated tonic stimulatory effect of endogenous dopamine on psychomotor activity. Indeed, a recent study in inducible D2R knockout adult mice that obviated developmental compensations reported that the loss of D2R was associated with severe hypolocomotion and catalepsy (Bello et al., 2017). Likewise, the spontaneous locomotor activity of A2AR^-/-^ mice was also significantly reduced, as previously reported in the A2AR C57BL/6 mouse (Yang et al., 2009). Since pharmacological blockade leads to significant locomotor activation (see below and Karcz-Kubicha et al., 2003; Orrú et al., 2011a), the reduced activity of A2AR^-/-^ mice indicates the development of neuroadaptations that counteract the loss of an A2AR-mediated tonic inhibitory effect of endogenous adenosine on psychomotor activity. We also assessed sensorimotor processing of A2AR^-/-^ and D2R^-/-^ CD-1 mice by monitoring pre-pulse inhibitory responses (PPI) (Taura et al., 2017). As compared with WT mice, D2R^-/-^ mice did not show significant differences, while A2AR^-/-^ mice showed a significantly reduced PPI as previously reported (Wang et al., 2003; Moscoso-Castro et al., 2016), demonstrating a significant dependence on A2AR, but not D2R, signaling for a normal PPI. We also evaluated drug-induced catalepsy in A2AR^-/-^ and D2R^-/-^ mice. Our results showed that haloperidol-induced catalepsy was abolished and partially but significantly reduced in D2R^-/-^ and A2AR^-/-^ mice, respectively, as compared with WT mice (Taura et al., 2017), which is in agreement with previous work (Usiello et al., 2000; Chen et al., 2001; El Yacoubi et al., 2001). The results support the dependence on A2AR signaling in the catalepsy induced by pharmacological blockade of D2R, which would agree with the existence of the tonic inhibition of A2AR signaling by endogenous dopamine driven by the canonical interaction in the A2AR-D2R heteromer. Neuroadaptations occurring in the A2AR^-/-^ mouse should explain the partial effect of genetic blockade of A2AR on D2R antagonist-induced catalepsy, which contrasts with the very effective blockade with A2AR antagonists (see below and Kanda et al., 1994; Shiozaki et al., 1999; Morelli and Wardas, 2001). We also assessed catalepsy induced by the A2AR agonist CGS 21680 (Ferré et al., 1991a; Kanda et al., 1994; Hauber and Münkle, 1997) in A2AR^-/-^ and D2R^-/-^ mice. As expected, CGS 21680 failed to induce catalepsy in A2AR^-/-^ mice, but its effect was partially but significantly reduced in D2R^-/-^ mice (Taura et al., 2017). Again, these results might reflect a functional antagonism related to neuroadaptations associated with genetic D2R blockade, which would tend to counteract the loss of the D2R-mediated tonic stimulatory effect of endogenous dopamine on psychomotor activity.

**FIGURE 6:**
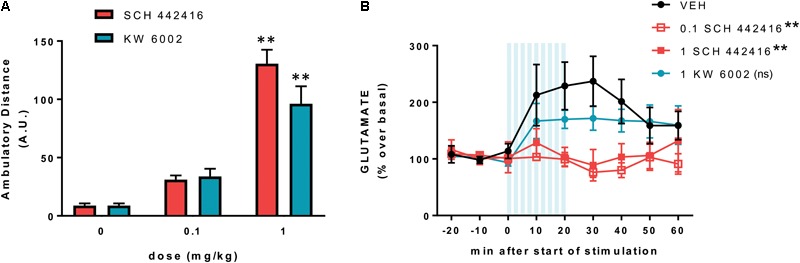
Preferential presynaptic profile of SCH 442416 in C57BL/6 mice. **(A)** SCH 442416 shows similar potency and efficacy to KW 6002 at producing locomotor activation. Locomotor activity was measured in an open field arena as described elsewhere (Bonaventura et al., 2015); animals were injected intraperitoneally (i.p.) with vehicle (saline with 10% DMSO and 10% Tween-80) and the indicated concentrations of SCH 442416 or KW 6002 and the locomotor activity was measured for 2 h in activity chambers with 42.0 cm × 42.0 cm open fields (Coulbourn Instruments); values are mean ± SEM of the traveled distance (arbitrary units, A.U.); two-way ANOVA with Newman–Keuls *post hoc* test did not demonstrate significant differences between the two A2AR antagonists and, for both drugs, it only showed significant differences with the dose of 1 mg/kg as compared to the corresponding vehicle-treated groups (^∗∗^*p* < 0.01 compared to vehicle; *n* = 8–11). **(B)** Optogenetic-microdialysis experiments were performed as described elsewhere (Bonaventura et al., 2017); briefly, C57BL/6 mice received a unilateral injection of an AAV encoding ChR2 (ChR2/H134R) fused to EYFP under control of the CaMKIIa neuronal promoter [AAV-CaMKIIa-hChR2(H134R)-EYFP] in the motor cortex. One month later, an optogenetic-microdialysis probe (Quiroz et al., 2016; Bonaventura et al., 2017) was implanted in the dorsal striatum, and glutamate in the dialysate was measured at 10-min intervals before, during, and after optogenetic stimulation of the corticostriatal terminals. Vehicle (black plot, see above) or the indicated doses of SCH 442416 (red plot) or KW 6002 (blue plot) were administered (i.p.) 10 min before the start of the stimulation. Values (in % over basal) represent mean ± SEM, normalized to the mean of the concentration of GLU present in the three samples preceding stimulation; one-way ANOVA with Newman–Keuls *post hoc* test showed a significant decrease of the transformed values (area under the curve, data from min 0 to min 60) from both groups treated with SCH 442416 (1 and 0.1 mg/kg), but not from the group treated with KW 6002, compared to the vehicle group (^∗∗^*p* < 0.01 compared to vehicle; ns, non-significant; *n* = 7–8).

We then reevaluated the effect of SCH 442416 on locomotion, PPI and drug-induced catalepsy in WT, but also in A2AR^-/-^ and D2R^-/-^ mice. In WT CD-1 mice, SCH 442416 produced a significant and effective locomotor activation at 1 mg/kg (i.p.) (Taura et al., 2017), a dose three times lower than the minimal effective dose in Sprague-Dawley rats (Orrú et al., 2011a). As expected, SCH 44241 was unable to alter the locomotor activity in A2AR^-/-^ mice and it only moderately, but significantly, increased the activity in D2R^-/-^ mice (Taura et al., 2017). The decrease in the effect of the A2AR antagonist in D2R^-/-^ mice would agree with a dependence on D2R signaling in the locomotor activation induced by pharmacological blockade of A2AR, due to the tonic inhibition of D2R signaling by endogenous adenosine driven by the allosteric interaction in the A2AR-D2R heteromer. In agreement with the dependence on A2AR for PPI, SCH 442416 (at the minimal dose of 3 mg/kg, i.p.) induced a blockade of PPI in WT mice (Taura et al., 2017). This is also in agreement with a previous study in rats with intracranial infusion of another A2AR antagonist (MSX-3) in the NAc (Nagel et al., 2003). SCH 442416 was obviously ineffective on the already disrupted PPI in A2AR^-/-^ mice, but its disruptive effect was reduced in D2R^-/-^ mice (Taura et al., 2017). This could be related to the competing effect of endogenous adenosine by the released tonic inhibition of A2AR signaling by endogenous dopamine driven by the canonical interaction in the A2AR-D2R heteromer. Finally, SCH 442416 significantly reduced haloperidol-induced catalepsy, as previously reported for other A2AR antagonists (Kanda et al., 1994; Shiozaki et al., 1999; Morelli and Wardas, 2001), but with a higher minimal dose than the one needed to produce locomotor activation (3 vs. 1 mg/kg, i.p., respectively; Taura et al., 2017). To confirm the preferential pre- vs. postsynaptic profile of SCH 442416 in mice, we also performed dose-response experiments in C57BL/6 mice on locomotor activity and counteraction of corticostriatal glutamate release using a recently introduced optogenetic-microdialysis technique (Quiroz et al., 2016; Bonaventura et al., 2017). Different to previous experiments in rats, SCH 442416 showed the same potency and efficiency as the selective A2AR antagonist KW 6002 at eliciting locomotor activation. Both drugs produced significant activation at 1 mg/kg (i.p.) but were inefficient at 0.1 mg/kg (**Figure 6A**). At this moment, we do not have an explanation for the lower potency and efficacy of SCH 442416 in rats as compared to mice. On the other hand, SCH 442416 was able to block optogenetically induced striatal glutamate release at 0.1 mg/kg, while KW 6002 was ineffective at 1 mg/kg (**Figure 6B**). This confirmed the experimental findings in rats, demonstrating a predominant striatal presynaptic and postsynaptic A2AR blocking properties of SCH 442416 and KW 6002, respectively (Orrú et al., 2011a).

Altogether, the results with genetic and pharmacological blockade of A2AR and D2R agree with a main role of A2AR-D2R heteromers in the striatopallidal neuron in conveying locomotor activation and PPI disruption induced by A2AR antagonists and D2R agonists and catalepsy mediated by A2AR agonists and D2R antagonists. More specifically, they also agree with A2AR-D2R heteromers in striatopallidal neurons mediating all postsynaptic pharmacological effects of SCH 442416, locomotor activation, blockade of PPI and counteraction of D2R antagonist-induced catalepsy. As shown in the scheme of **Figure 3A**, the A2AR-D2R heteromer explains the qualitatively different behavioral outputs depending on direct A2AR-Golf-AC-PKA-mediated increase in excitability or indirect D2R-Gi-PLC-PP2B-mediated disinhibition of the excitability of the striatopallidal neuron, leading to catalepsy and PPI (more related to the direct activation of the PKA-DARPP-32-CREB signaling; Bateup et al., 2010; Berger et al., 2011) or just psychomotor depression, respectively. In fact, it is well known that catalepsy, with its rigidity component, is not qualitatively equivalent to a high degree of locomotor depression. Finally, and as mentioned before, depending on the intracellular concentrations of Ca^2+^, A2AR and D2R activation and co-activation lead to differential MAPK and Elk-1 activation, with implications for gene expression and synaptic plasticity (**Figure 3A**).

## A2AR-D2R Heteromer-Mediated Control of the Ventral Vs. Dorsal Striatopallidal Function and Implications for Neuropsychiatric Disorders. ‘Apathy’ Vs. ‘Akinesia’

Dysfunction of the central dopamine system is involved in a variety of disorders, including Parkinson’s disease, schizophrenia, and substance use disorders (SUD). The functional separation of striatal compartments in ventral, rostral-dorsal and caudal-dorsal striata allows a more syndromic sub-classification of those disorders with potentially significant new therapeutic approaches. Parkinson’s disease and non-human primate models of Parkinson’s disease provide the clearest illustration. The cardinal motor symptoms of Parkinson’s disease, bradykinesia, rigidity and tremor (Jankovic, 2008), have been classically attributed to dysfunction of the skeletomotor system, the brain circuits involved in the execution and coordination of body movements. Contemporary theories embracing parallel cortical-striatal-thalamic-cortical circuits in the pathogenesis of this disorder emphasize the particular involvement of the “motor circuit,” which includes motor cortical areas (DeLong and Wichmann, 2015). In fact, in Parkinson’s disease, dopamine cell degeneration tends to occur initially and predominantly in the lateral part of the SNpc, which projects mainly to the caudal-dorsal striatum. Thus, there is a predominant deficit of the more “automatic” vs. “volitional” action skills and most sequential psychomotor responses need to be performed with full attention (Kim and Hikosaka, 2015). Nevertheless, with more advanced stages of Parkinson’s disease the function of the more rostral striatum becomes also compromised, with deficits in “volitional” actions skills (Kim and Hikosaka, 2015). With further (or preferential) ventral degeneration of the dopamine mesencephalic nuclei (VTA) we move to the pathology of the ventral striatum, to apathy (Tremblay et al., 2015), as it has also been demonstrated experimentally in the non-human primate (Brown et al., 2012; Tian et al., 2015).

Initial studies on the psychomotor-activating effects of caffeine or selective A2AR antagonists dealt with general locomotor activity and were translationally applied to the treatment of akinesia in Parkinson’s disease (see above and Müller and Ferré, 2007; Morelli et al., 2009; Armentero et al., 2011). Those initial studies implicitly considered A2AR-D2R heteromers in the dorsal striatum, but a large number of studies indicated that not only dorsal but also ventral striatopallidal neurons express A2R and A2AR-D2R heteromers (Ferré et al., 1994; Ferré, 1997; Hauber and Münkle, 1997; Pinna et al., 1997; Svenningsson et al., 1997; Ishiwari et al., 2007). More recent studies have also analyzed the effect of caffeine and A2AR antagonists on more specific reward-oriented behaviors, showing that they can increase the responsiveness to food-related stimuli, sucrose solutions, stimuli that elicit maternal behavior and self-administration (Pereira et al., 2011; Randall et al., 2011; Sheppard et al., 2012; Nunes et al., 2013; Lazenka et al., 2015). The work by Salamone’s group has specifically addressed the role of adenosine and A2AR in effort-related choice behavior. Direct administration of A2AR agonists in the NAc altered effort-related choice behavior in a manner closely resembling the effects of interference with ventral striatal dopamine neurotransmission, decreasing the degree of responsiveness (“effort”) to reward-associated stimuli. Furthermore, A2AR antagonists reversed the effort discounting effects of D2R antagonists (Salamone et al., 2012; Nunes et al., 2013).

Clinically, apathy has been defined as “a syndrome consisting of loss of motivation not attributable to disturbances in emotion, intellect or consciousness” (Marin, 1991). However, it is becoming obvious that apathy is a multifaceted concept that includes dissociable constructs that should correspond to dissociable neurobiological correlates (Sinha et al., 2013). We hypothesize that some if not all those dissociable correlates correspond to corticostriatal circuits involving the different functional striatal compartments and their “Go” and “NoGo” pathways. In fact, attuned with the role of dopamine in reward-associated behavior in all striatal compartments, recent studies even allow conceptualizing Parkinson’s disease bradykinesia in a motivational framework (Mazzoni et al., 2007; Chong et al., 2015). Nevertheless, as defined clinically, apathy is a common non-motor symptom of Parkinson’s disease (den Brok et al., 2015) that correlates negatively with dopamine innervation in the ventral striatum (Remy et al., 2005; Chaudhuri et al., 2006; Brown et al., 2012). In fact, a deficit in the dopamine modulation of the ventral striatum should translate, first, in a deficit in responsiveness, with a global inability to respond to reward- and punishment-associated stimuli (attuned with the “whether to respond” vs. “how to respond” functions of ventral vs. dorsal striatum). Second, it should lead to dysfunction of reward valuation, in alterations (increase) in DD, ED and LPD (attuned with the ventral striatum as forming part of corticostriatal circuits involved in reward valuation tasks). Indeed, non-medicated patients with Parkinson’s disease have shown increases in DD and ED (Al-Khaled et al., 2015; Chong et al., 2015).

Interestingly, apathy is also a major negative symptom of schizophrenia, classically considered as a disorder associated with central hyperdopaminergic tone. Several studies have found evidence for selective dysfunction of the ventral striatum in schizophrenia, specifically hypoactivation with reward-associated stimuli (Simon et al., 2010, 2015; Strauss et al., 2015; Kirschner et al., 2016). Ventral striatal activation during reward anticipation was in fact found to be selectively and inversely correlated with apathy but not with other negative symptoms (Simon et al., 2010; Kirschner et al., 2016). Two additional findings give a clue for the mechanisms of apathy in schizophrenia, which seem to be dopamine-independent or at least not related to a decrease in the dopamine tone. First, there is a reduced functional connectivity between the orbito-frontal cortex (OFC) and the ventral striatum (Simon et al., 2015); second, there is consistent evidence that schizophrenic patients suffer from selective deficits in learning from positive outcomes, with intact learning from negative outcomes (Strauss et al., 2015). Therefore, the apathetic schizophrenic patient seems to have a selective decreased activation of the “Go” pathway, a reduction in the ratio of activation of “Go” vs. “NoGo” pathways secondary to impaired cortical-ventral striatal connectivity (Strauss et al., 2015). A similar situation would also be present in the patient with SUD, a decreased “Go”/“NoGo” pathway activation, also with reduced ventral striatal activation to reward stimuli (which can basically only be activated by the addictive drugs) (Volkow et al., 2011). Apathy is a well-known symptom in SUD, although it has been scarcely addressed experimentally (Verdejo-García et al., 2006; Verdejo-García and Pérez-García, 2008; Gjini et al., 2014). The SUD patient is motivated to procure the drug but tends to be withdrawn and apathetic when exposed to non-drug-related activities (Verdejo-García et al., 2006). In this case, however, the pathogenesis seems to follow from an initial reduction in D2R density (maybe with a concomitant relative increase of A2AR which would not be opposed by D2R forming heteromers), leading to an increased activity of the ventral striatopallidal neuronal function, of the “NoGo” pathway. The tonic decrease in feedback activation of the ventromedial prefrontal cortex, orbitofrontal cortex and anterior cingulate cortex leads to additional dysfunction of the decision-making cortical-ventral striatal circuits (Volkow et al., 2011; Belcher et al., 2014). These changes lead to a similar situation than the non-motor symptoms in patients with Parkinson’s disease, to apathy and choice impulsivity, as demonstrated by several studies indicating increase DD in patients with SUD (Belcher et al., 2014; Hamilton et al., 2015). In summary, for all types of apathy, the relative increase in the ventral striopallidal vs. striatonigral neuronal function should benefit from the treatment with A2AR antagonists, targeting A2AR-D2R heterotetramer-AC5 complexes.

## Conclusion

A significant amount of experimental and clinical evidence demonstrates that A2AR and D2R localized in the ventral and dorsal striatopallidal neurons cannot be considered anymore as single functional units, but as forming part of complexes of the A2AR-D2R heterotetramer-AC5 complexes, which exert a fine-tuned integration of adenosine and dopamine neurotransmission. The current accumulated knowledge of the biochemical properties of the A2AR-D2R heteromer offer new therapeutic possibilities for Parkinson’s disease, schizophrenia, SUD and other neuropsychiatric disorders with dysfunction of dorsal or ventral striatopallidal neurons. More generally, this knowledge implies we should modify classical views of GPCR physiology and pharmacology and include GPCR heteromers as main targets for drug development. The understanding of the biochemical properties of GPCR heteromers specifically localized in neuronal elements that form part of neuronal circuits involved in the pathophysiology of specific neuropsychiatric disorders should provide new selective pharmacological approaches with less unwanted side effects.

## Ethics Statement

All animals used in the study were maintained in accordance with the guidelines of the National Institutes of Health (NIH) Animal Care, and the animal research conducted to perform this study was approved by the NIDA IRP Animal Care and Use Committee (protocols #12-BNRB-73, #15-BNRB-73, and #12-MTMD-2).

## Author Contributions

JB, WZ, CH-S, and KT performed the experiments and analyzed the data. SF, JB, KT, AK, VC, DL, and DZ designed the experiments. SF, JB, JT, CQ, N-SC, EM, VC-A, AK, DT, GN, AC, LP, CL, CWD, NV, VC, FC, DL, and DZ wrote the manuscript.

## Conflict of Interest Statement

The authors declare that the research was conducted in the absence of any commercial or financial relationships that could be construed as a potential conflict of interest.
